# Concurrent development of facial identity and expression discrimination

**DOI:** 10.1371/journal.pone.0179458

**Published:** 2017-06-15

**Authors:** Kirsten A. Dalrymple, Matteo Visconti di Oleggio Castello, Jed T. Elison, M. Ida Gobbini

**Affiliations:** 1 Institute of Child Development, University of Minnesota, Minneapolis, MN, United States of America; 2 Australian Research Council Centre of Excellence in Cognition and its Disorders, University of Western Australia, Crawley, Australia; 3 Department of Psychological and Brain Sciences, Dartmouth College, Hanover, NH, United States of America; 4 Department of Experimental, Diagnostic and Specialty Medicine (DIMES), University of Bologna, Bologna, Italy; Bournemouth University, UNITED KINGDOM

## Abstract

Facial identity and facial expression processing both appear to follow a protracted developmental trajectory, yet these trajectories have been studied independently and have not been directly compared. Here we investigated whether these processes develop at the same or different rates using matched identity and expression discrimination tasks. The Identity task begins with a target face that is a morph between two identities (Identity A/Identity B). After a brief delay, the target face is replaced by two choice faces: 100% Identity A and 100% Identity B. Children 5-12-years-old were asked to pick the choice face that is most similar to the target identity. The Expression task is matched in format and difficulty to the Identity task, except the targets are morphs between two expressions (Angry/Happy, or Disgust/Surprise). The same children were asked to pick the choice face with the expression that is most similar to the target expression. There were significant effects of age, with performance improving (becoming more accurate and faster) on both tasks with increasing age. Accuracy and reaction times were not significantly different across tasks and there was no significant Age x Task interaction. Thus, facial identity and facial expression discrimination appear to develop at a similar rate, with comparable improvement on both tasks from age five to twelve. Because our tasks are so closely matched in format and difficulty, they may prove useful for testing face identity and face expression processing in special populations, such as autism or prosopagnosia, where one of these abilities might be impaired.

## Introduction

Face processing is a complex cognitive ability that we rely on to process important information about others, such as identity, emotional expression, direction of attention, sex, and age. Early theoretical models of face processing have suggested that some of these abilities operate independently [1–3]. For example, one particularly influential model of face processing [1] proposed a clear separation between what was referred to as ‘expression analysis’ and components dedicated to identity recognition called ‘face recognition units’ and ‘person identity nodes’. Complementing this model, Haxby and colleagues [2, 4] proposed a distributed human neural system for face processing that assigned the processing of changeable aspects of faces (such as for facial expression) to the Superior Temporal Sulcus (STS), and the processing of invariant aspects of faces (such as for facial identity) to the Lateral Fusiform Gyrus. These models propose that the separation between the processing of facial identity and expression occurs early, and that these processes remain separate. Electrophysiological and neuropsychological evidence supports these models. For example, repetitive transcranial magnetic stimulation to the right occipital face area disrupts both identity and expression processing whereas stimulation of the right posterior superior temporal sulcus and somatosensory cortex selectively disrupts expression processing and not identity processing [5–7]. In addition, some brain damaged individuals with impaired expression recognition have normal identity recognition [8–10], and vice versa [11, 12]. Cases of developmental prosopagnosia, characterized by severe face identity recognition deficits, can also have normal facial expression recognition, suggesting that these abilities can develop separately [13, 14]. There is evidence that these abilities may separate at a young age: a 5-year-old boy with severe prosopagnosia nevertheless demonstrated normal facial expression processing [15].

In contrast to the models that suggest independent processing of identity and expression information, other models suggest a complex interaction between the two, positing that rather than operating completely autonomously, there may be a single representation system that processes both identity and expression [16, 17]. Supporting this interactive view, there is evidence of an asymmetric relationship between these processes such that identity information interferes with expression processing, but not the other way around (i.e. participants can ignore task-irrelevant expression information and selectively attend to identity information in a speeded identity sorting task, but they cannot ignore task-irrelevant identity information during a speeded expression sorting task) [18, 19, though see 20 for findings showing that expression can interfere with identity judgments]. Functional Magnetic Resonance Imaging (fMRI) findings also show that the Fusiform Face Area, traditionally associated with the processing of facial identity [2], is also activated when processing facial expression, and responds to irrelevant changes to expression when attention is directed to facial identity [21]. Additionally, some groups question the neuropsychological dissociation between identity processing and expression processing in individuals with face recognition deficits [16]. They argue that although individuals with prosopagnosia often report intact expression recognition, careful testing can reveal deficits in expression processing, albeit often less severe than the identity processing difficulties that define prosopagnosia. Ultimately, the theoretical debate about the independence of identity and expression processing continues.

Normal face processing appears to follow a protracted developmental course. Improvements in facial identity recognition have been reported from early childhood (e.g. as young as 5 years) through adolescence (e.g. 16 years) [22–28], even when controlling for general cognitive factors, such as IQ [22]. Online testing of more than 60,000 people showed peak face recognition performance well into adulthood, at age 32 [29, 30]. It has been argued that some of these age-related improvements in face processing can be explained by general cognitive development (e.g. improved attentional skills) rather than the development of face specific processes [31]. However, this view did not take into account the possibility that different aspects of face identity processing may develop at different rates. In fact, it was recently discovered that the ability to *discriminate* the identity of faces that are presented simultaneously develops at a different rate than the ability to *remember* the identity of a face [28]. This suggests that, within identity processing, face perception and face memory are dissociable and follow different developmental trajectories.

With regards to the development of expression processing, studies in children from preschool to adolescence show development of facial expression processing from 3.5 years to 15 years [32–36]. More recent studies indicate that children can recognize happy expressions at very subtle intensities early on (i.e. by 5 years), but that sensitivity to other expressions continues to develop until 10 years, and beyond [37, 38]. Despite the converging evidence suggesting an extended developmental timeline, each of these studies have examined expression recognition in a different age range and it is difficult to compare findings across studies because of methodological differences, such as different stimuli and tasks [39].

While it appears that both identity recognition and expression recognition follow a lengthy developmental trajectory, these trajectories have not been directly compared. Recent MRI studies provide some insight into what such a comparison might reveal. Face selective areas within the fusiform gyrus, which is implicated in face identity recognition, increase in size [40, 41] (and density, see [42]) from childhood to adulthood. In contrast, face selective areas in the Superior Temporal Sulcus, which is implicated in the processing of gaze information and social communication cues, do not appear to change in size during this time [40]. This differential development of these brain areas predicts that in matched tasks of identity and expression processing, an interaction between age and task could emerge. Specifically, slow growth of face-selective fusiform areas may reflect slower behavioral improvement in face identity processing behavior, while an adult-sized STS in childhood may reflect a more rapid development of functions associated with the STS, such as expression processing.

In the present study we aimed to test this prediction by directly comparing the development of identity and expression processing. We created tasks of facial identity and expression discrimination that are matched in format and difficulty to assess the development of these abilities in children 5-12-years-old. Both tasks present an ambiguous target face that is a morph between two faces that vary on the dimension of interest. After a brief delay, the target face is replaced by two choice faces, and the child is asked to pick the choice face that is most similar to the target on the task dimension. If we find an interaction between task and age, such that performance improves more quickly with age for one task than the other, this would support theories that suggest that these abilities operate independently. In contrast, if we find similar improvement with age for the two tasks, this could support theories that posit that identity and expression processes may rely on shared mechanisms. These matched tests of face identity and expression discrimination provide the first direct comparison of the developmental trajectories of these abilities. Sensitive tests of these related but dissociable face processing functions can provide indices of deviations from normal developmental trajectories in children with disorders such as developmental prosopagnosia or autism.

## Method

### Participants

Participants (n = 136, 63 = females, 130 = right handed) between the ages of 5-12-years (mean = 8.3; SD = 2.3) were recruited by email or over the phone through the research participant registry at the Institute of Child Development at the University of Minnesota. We chose this age range because we wanted to examine pre-adolescent school-age children. The data from nine participants were not included in the analysis because they did not complete one or both tasks, resulting in a sample of 127 children (Table 1). Upon arrival, the experimenter explained that the purpose of the study was to assess face processing in typically developing children. After the study was explained in detail, parents signed permission forms, and children who were 8-years-old or older signed assent forms to confirm their willingness to volunteer in the study. Children completed a series of tasks for this and other studies. Breaks were given between tasks when requested. Testing took less than 1 hour. Children were compensated for their participation. This study was approved by the Institutional Review Board at the University of Minnesota.

**Table 1:** Participants by age and gender.

Age (years)									
	5	6	7	8	9	10	11	12	Total
**Recruited**									
**Male**	11	11	8	12	9	8	7	7	73
**Female**	9	9	7	6	7	7	10	8	63
**Total**	**20**	**20**	**15**	**18**	**16**	**15**	**17**	**15**	**136**
Excluded*									
**Male**	3	2	0	1	0	0	0	0	6
**Female**	2	0	0	0	1	0	0	0	3
**Total**	**5**	**2**	**0**	**1**	**1**	**0**	**0**	**0**	**9**
**Final analysis**									
**Male**	8	9	8	11	9	8	7	7	67
**Female**	7	9	7	6	6	7	10	8	60
**Total**	**15**	**18**	**15**	**17**	**15**	**15**	**17**	**15**	**127**

*Participants were excluded for not completing one or both tasks

### Tasks

**Fig 1:**
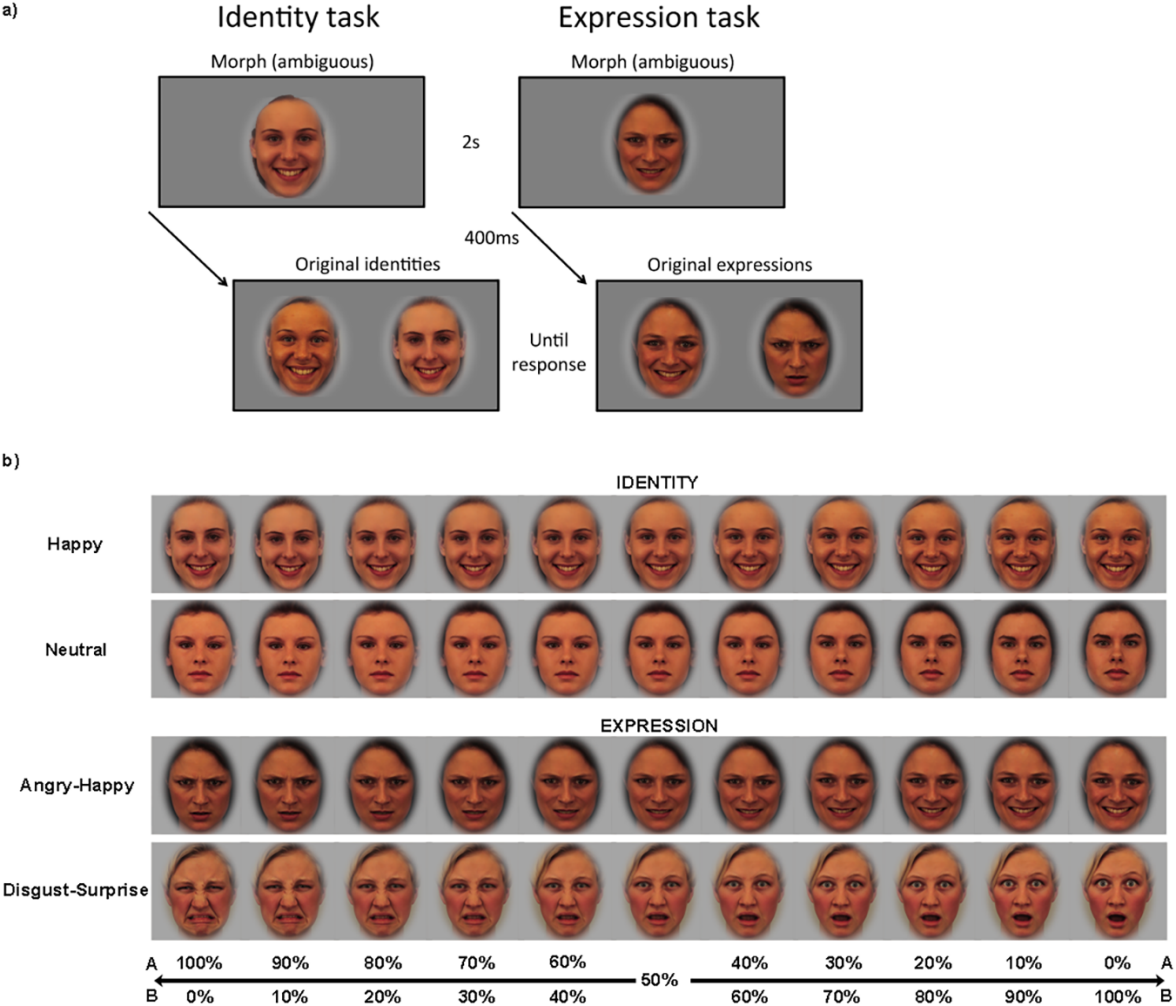
Experimental paradigm. a) Format of identity and expression discrimination tasks. b) Example morph continuums for identity and expression tasks.

We developed matched tasks of facial identity and expression discrimination (Fig 1A). The Identity task begins with the presentation of a target face (2s) that is a morph between two identities (Identity A/Identity B). After a delay (400ms), the target face is replaced by two choice faces: 100% Identity A and 100% Identity B. A total of 4 male and 4 female identities were used to create 4 morph continuums (2 male continuums, 2 female continuums, Fig 1B). One male and one female morph continuum had happy facial expressions and the others had neutral facial expressions. The child was asked to pick the choice face that is most similar to the target identity by pressing an arrow key (left arrow = face on the left, right arrow = face on the right). The experimenter emphasized that the child should do his or her best to choose the “correct” face and not be concerned about speed. The children were not told that there was no correct answer in the 50% morph trials. The Expression task is matched in format and difficulty to the Identity task, except the targets are morphs between two expressions (Angry/Happy, or Disgust/Surprise). In each trial the target and choice faces were of the same identity. Two male and two female identities were used (i.e. one male and one female Angry/Happy, one male and one female Disgust/Surprise). The child was asked to indicate by key press which choice face has the expression that is most similar to the target expression, again emphasizing that the child should try to choose the “correct” face, and not be concerned about speed.

Before beginning, children were given eight practice trials for each task. The experiment itself was broken down into eight randomly administered blocks of trials: 4 blocks Identity, 4 blocks Expression. Each of the 11 morph weights (i.e. 0%, 10%. . . 90%, 100%) appeared twice per block, in random order, using images from each of the different morph continuums for that task. Thus, there were 22 trials per block, for a total of 176 trials. Total test time took between 10–15 minutes.

### Stimuli

Stimuli were adult male and female faces from the Karolinska Directed Emotional Faces database [43]. Identities were paired for morphing based on subjective similarity of basic characteristics such as hair color, face shape, etc. to help ensure that morphs appeared natural. We chose angry, happy, disgusted, surprised, and neutral emotional expressions from the set of basic universal emotions [44]. Faces were 8.7° x 8.7° when viewed at 60cm. They appeared against a solid gray background and were revealed through 7.6° x 8.7° oval windows that covered ears and most of the hair. Morph faces were presented in the center of the screen. Choice faces were presented side by side, separated by a distance of 2.45°.

### Analysis

We first removed trials with response time that exceeded 2.5 SD from the within-subject average response time. Thus, for each subject we removed trials with response times that were too fast, possibly indicating anticipatory responding, or too slow, possibly due to inattention. Table 2 shows the percentage of trials removed for each Age and Task. To ensure that an equal number of trials were removed across ages and tasks, we ran a logit model on the status of the trial (rejected/not rejected), entering Age and Task (Expression and Identity), with their interaction, and testing their significance using a Type 3 Analysis of Deviance (as implemented in the R package *car* [45]). None of the effects were significant, indicating that the same number of trials were removed across Ages and Tasks: Age X^2^ (1) = 0.37, *p* = .54; Task X^2^ (1) = 0.002, *p* = .97; and Age x Task X^2^(1) = 0.004, *p* = .95.

**Table 2:** Percentage of trials removed for each task by age.

Age (years)								
	5	6	7	8	9	10	11	12
**Identity**	3.0	2.8	2.2	2.5	2.5	3.7	3.2	2.3
**Expression**	3.0	2.7	2.7	2.3	3.7	2.7	2.5	3.4

Trials with a reaction time exceeding 2.5 SD from the within-subject average reaction time were removed.

#### Accuracy

We characterized performance in the two tasks by computing a measure of accuracy. For this analysis, we first removed all trials at 50% morphing (because there was no correct answer for these trials). Next, for each face pair continuum we classified a trial as correct if the subject responded “A” when the presence of “face A” was more than 50%, or pressed “B” when the presence of “face A” was less than 50%. We fitted a single logit mixed model across subjects entering Age and Task (Expression and Identity) with their interaction as fixed effects, and subjects and type of continuum as random effects (random intercepts). Statistical significance of the individual terms was tested using a Wald’s test (type 3) as implemented in the package *car* in R [45].

#### Reaction time

Reaction time data were calculated using only correct trials as defined above. We fitted a single linear mixed effect model across all the subjects using Age and Task (Expression and Identity) as fixed effects with their interaction, and subjects and type of continuum as random effects (random intercepts). Statistical significance of the individual terms was tested using a Wald’s test (type 3) as implemented by the package *car* in R [45].

#### Psychometric curves

To characterize discrimination performance for each age group, we estimated a psychometric curve at the group level by fitting logit mixed effect models on the subjects' responses (face A or B) for each of the eight morph continua and each of the eight age groups. We entered subjects as a random effect (with random intercepts) and the percentage of morphing (0–100% in 10% steps, rescaled to -1 and 1 to allow convergence) as a fixed effect. We analyzed the steepness of the psychometric function by using the parameter estimate (slope) of the percentage morphing term, since this estimate is proportional to the steepness of the logistic function at the point of maximal inflection (i.e., where the first derivative is maximal). The steepness of the psychometric function is a proxy of how categorical the judgment is and larger slope indicates better performance (i.e. perfect observers would show a step function, always selecting face A if the morph contained more than 50% face A, and always selecting face B if the morph contained less than 50% face A).

We obtained 64 such slopes (8 age groups x 8 face pairs), divided the face pairs into expression and identity tasks, and ran a two-way ANOVA (Age x Task) on the slopes to estimate statistical significance. Each cell contained four samples, one for each face pair continuum. We tested significance for the main and interaction effects. We also ran a two-way ANOVA (Age x Expression type) to compare slopes for the two types of expression morphs (Angry/Happy vs. Disgusted/Surprised). Because of the small number of data points per cell, we consider this analysis exploratory (S1 Fig). All analyses were performed in R (version 3.2.2) using the package *lme4* (version 1.1–10) for the fitting of the logit models, and *ggplot2* (version 2.0.0) for plotting the results.

## Results

### Accuracy

**Fig 2:**
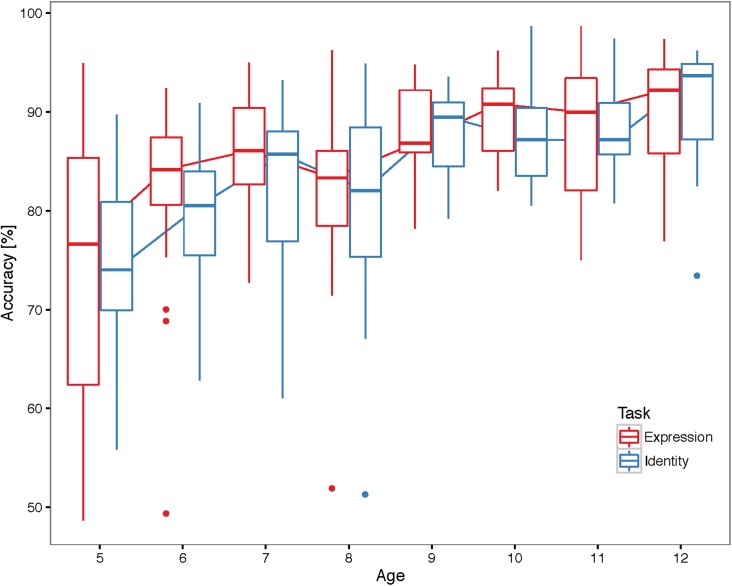
Participant accuracy. Boxplots showing average accuracy for subjects in each age group and task. Individual points represent outlier subjects (defined as those points exceeding 1.5 times the inter-quartile range). Accuracies increased with age, and were similar for both tasks.

**Fig 3:**
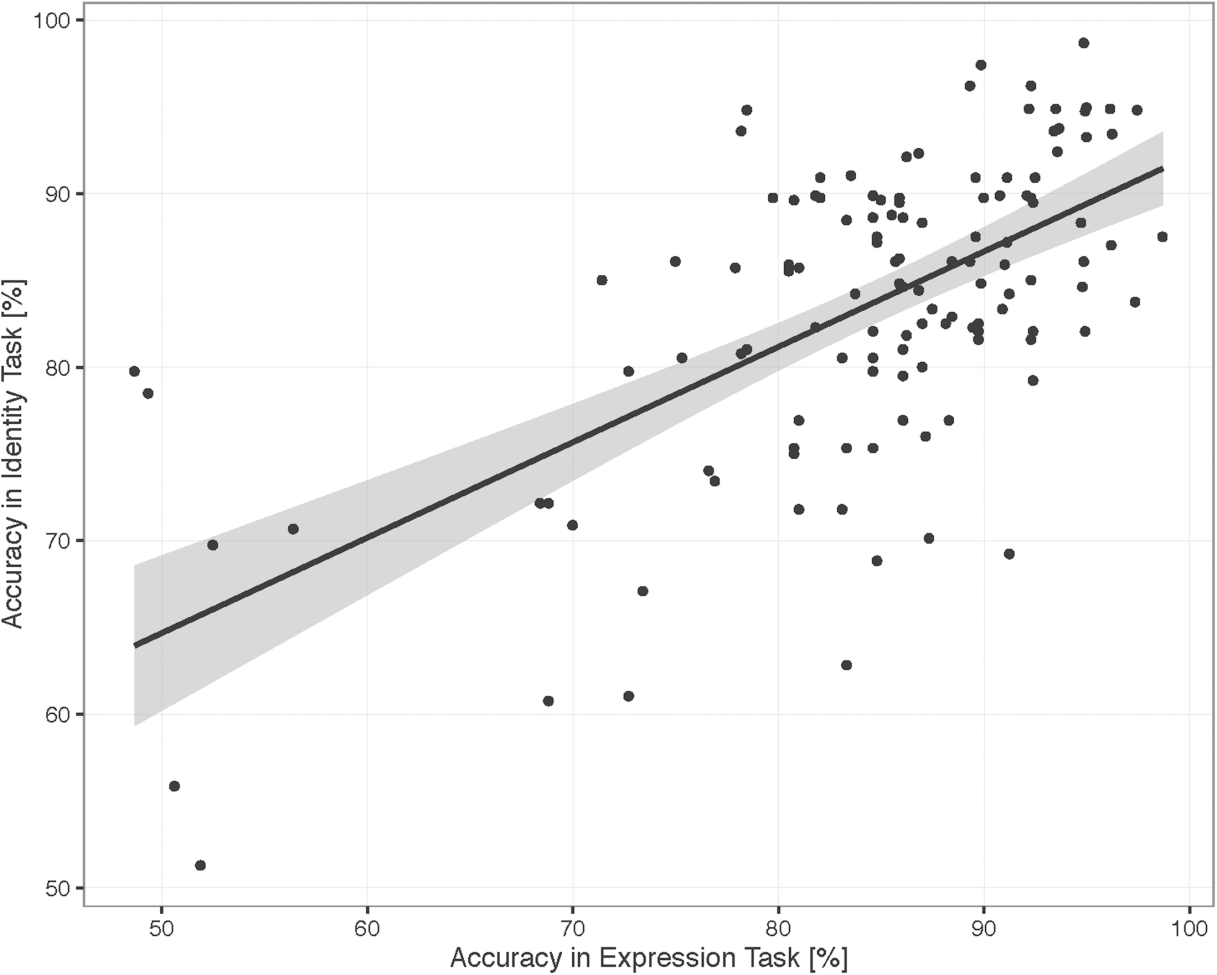
Correlation between identity and expression: Accuracy. Scatterplot illustrating correlation between participant accuracy on identity and expression tasks, in %. Data include all trials except those with 50% morphs, which do not have a correct response. Grey shading indicates 95% confidence interval.

Fig 2 shows the mean accuracies for each Age and Task (see S2 Fig for data plotted by individual). We found that the overall accuracy increased with age, Χ^2^(1) = 57.83,p < .001, and that the two tasks were matched in difficulty, as revealed by the non-significant main effect of Task, Χ^2^(1) = 0.44, *p* = 0.51 and non-significant interaction between Age and Task, Χ^2^(1) = 0.12, *p* = 0.73. Participant performance for one task was highly correlated with performance in the other task when controlling for Age, r_p_ = 0.51 partial correlation, n = 127, *p* < .001, Fig 3. See S3 Fig for correlations for each age.

### Reaction time

**Fig 4:**
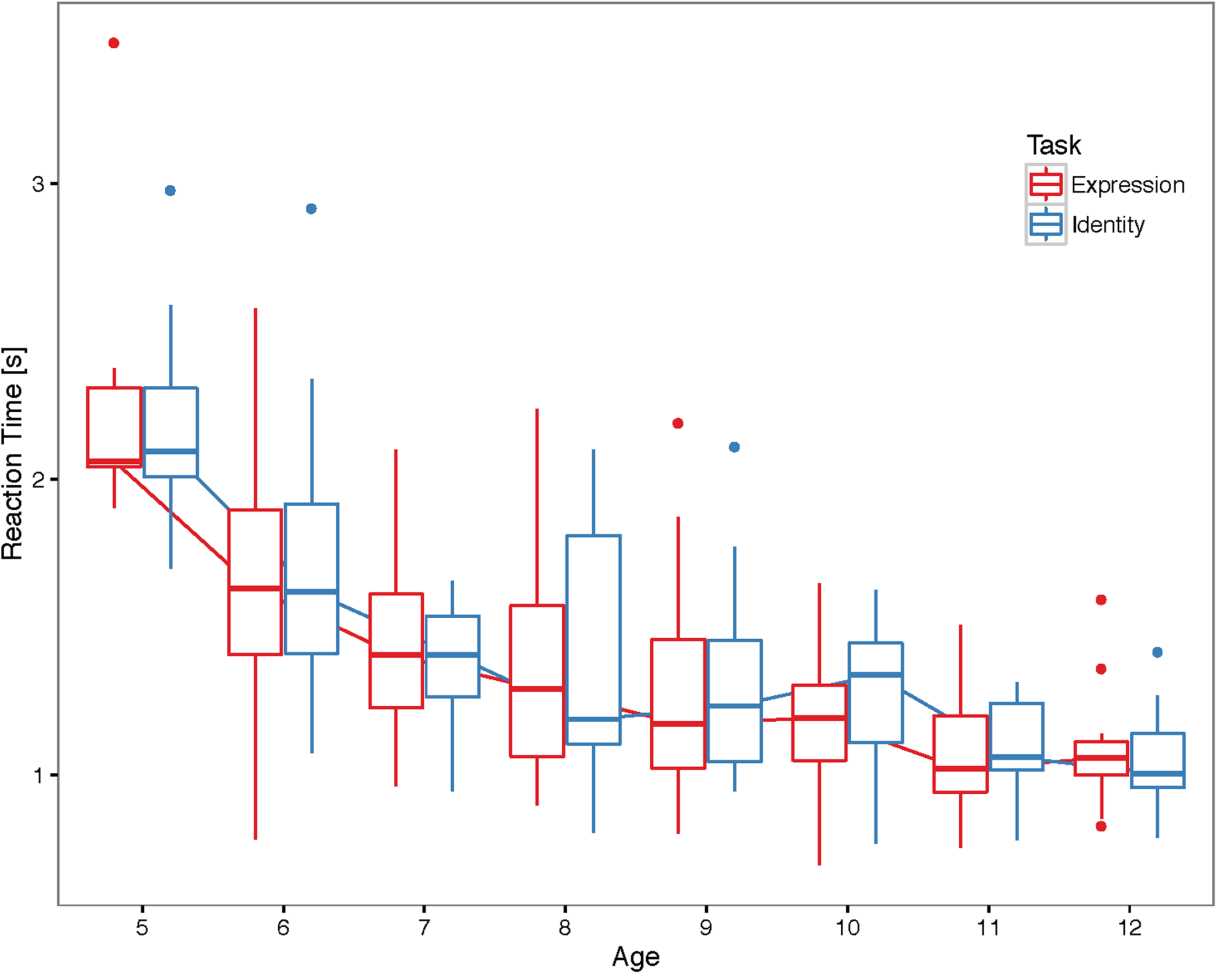
Participant reaction time. Boxplots showing average reaction time for subjects in each age group and task. Individual points represent outlier subjects (defined as those points exceeding 1.5 times the inter-quartile range).

**Fig 5:**
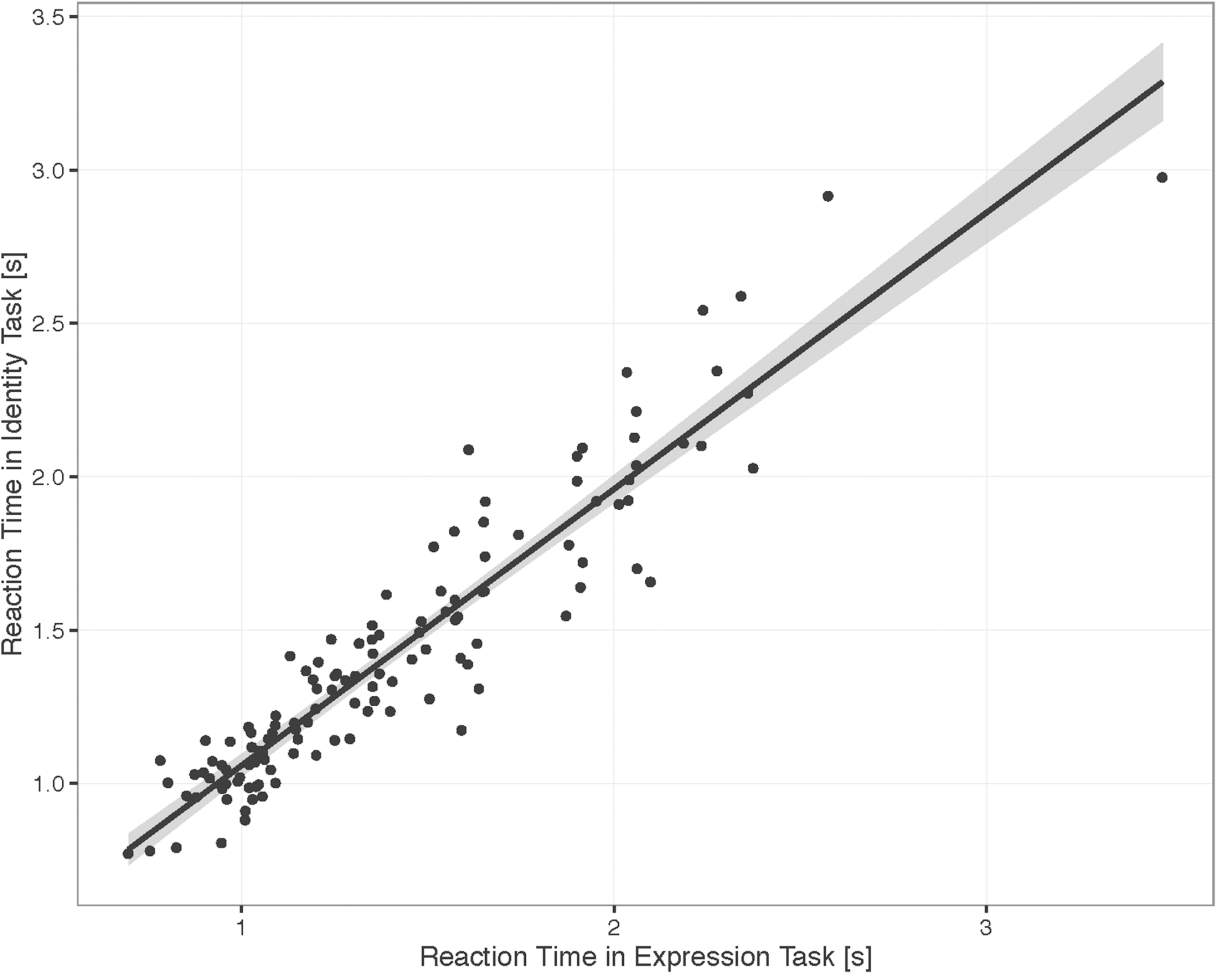
Correlation between identity and expression: Reaction time. Scatterplot illustrating correlation between participant reaction times on identity and expression tasks (in seconds). Data include only trials that were answered correctly (trials with 50% morphs are not included because they not have a correct response). Grey shading indicates 95% confidence interval.

Fig 4 shows the reaction times plotted according to Age and Task for correct trials only. We found a significant main effect of Age, Χ^2^(1) = 115.37, *p* < .001, showing that reaction times decreased with age. The main effect of Task was not significant, Χ^2^(1) = 0.01, *p* = 0.90, as well as the interaction, Χ^2^(1) = 0.40, *p* = 0.53. Participant reaction times in one task were highly correlated with reaction times in the other task when controlling for age, r_p_ = 0.88 partial correlation, n = 127, *p* < .001, Fig 5. See S4 Fig for correlations for each age.

### Psychometric curves

**Fig 6:**
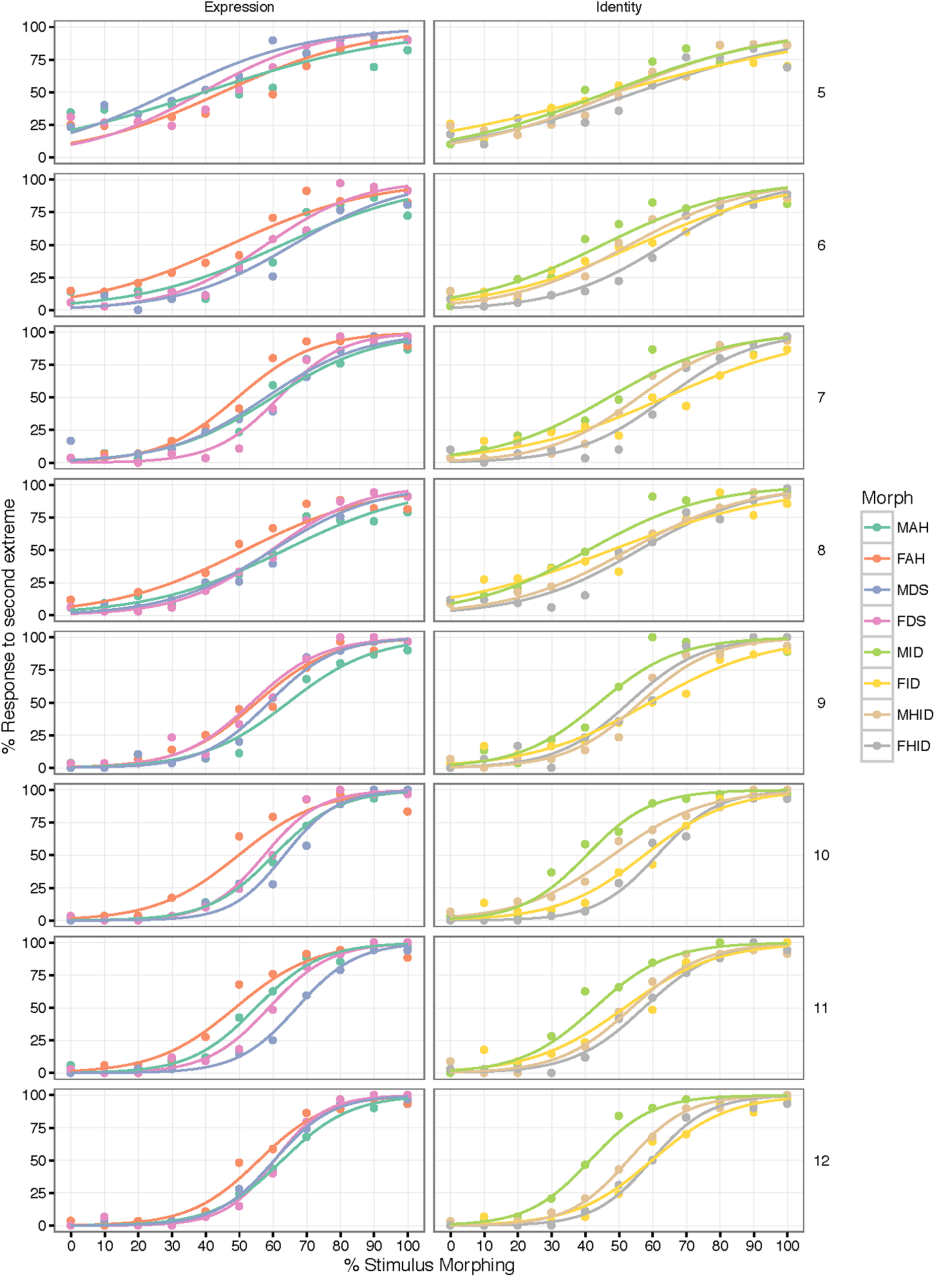
Psychometric curves for identity and expression tasks. Group-level psychometric curves for each age group and morph continuum, divided according to the task: Expression (left column) and Identity (right column). The x-axis maps the percentage of morphing from one extreme to the other (e.g. 30% MAH = Male Angry face with 30% Male Happy face); the y-axis maps the proportion of responses to the second extreme (for MAH, proportion of responses “Male Happy Face”). MAH = Male Angry to Happy; FAH = Female Angry to Happy; MDS = Male Disgust to Surprise; FDS = Female Disgust to Surprise; MID = Male Identity 1 to Identity 2 (neutral expression); FID = Female Identity 1 to Identity 2 (neutral expression); MHID = Male Identity 1 to Identity 2 (happy expression); FHID = Female Identity 1 to Identity 2 (happy expression).

**Fig 7:**
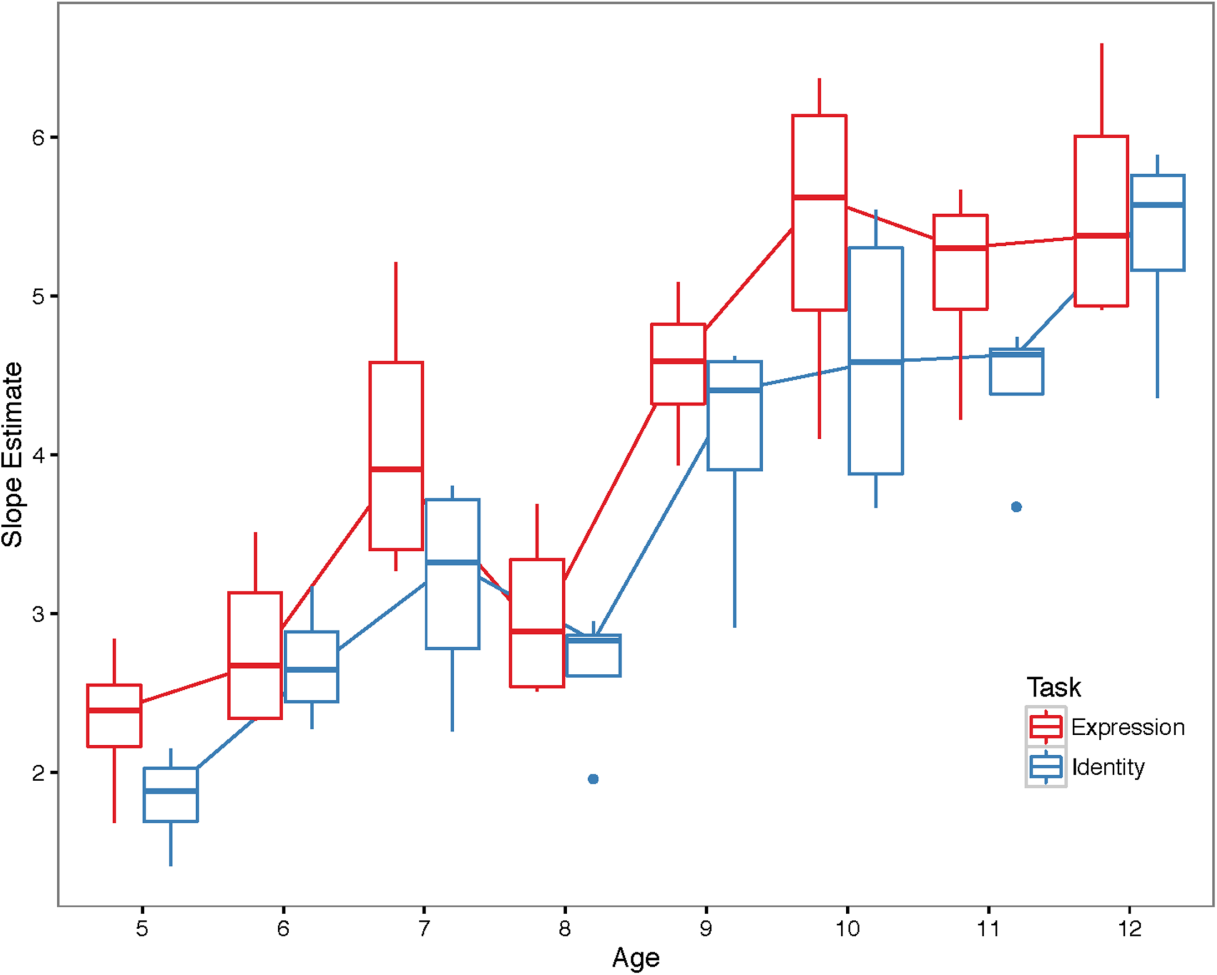
Slope estimates. Boxplots of the slope estimates for each age group and task. Individual points represent outliers (defined as those points exceeding 1.5 times the inter-quartile range).

Fig 6 shows the 64 models for each age group and face pair, divided according to Age and Task. Fig 7 shows the slopes of the curves according to Age and Task. Larger (steeper) slopes indicate better performance.

We found a significant main effect of Age, F(1, 60) = 137.10, *p* < .001, η_p_^2^ = 0.70, and a small, but significant main effect of Task, F(1, 60) = 7.69, *p* < .05, η_p_^2^ = 0.11, but no Age x Task interaction, F(1, 60) = 002, *p* = 0.89, η_p_^2^ ≅ 0.

Our analyses reveal that the slopes of the curves increase linearly with age (indicating better performance), and that overall, participants were able to discriminate expressions slightly better than identities. We quantified the effect of Age by averaging the slopes over the two tasks and computing the ratio between the slope at age 12 and the slope at age 5. We discovered that 12-year-olds showed slopes that were 2.63 steeper than 5-year-olds (Fig 7). We then quantified the effect of Task by computing the ratio between the average slope for expression and the slope for identity across all ages. This showed that the effect size of Task was only slight: slopes in the expression task were only 1.15 times steeper than in the identity task.

## Discussion

Whether face identity and expression are processed separately or by shared mechanisms is an ongoing topic of debate [5–14, 16, 18–21, 46]. The present study was designed to directly compare the development of these two face processing abilities, in tasks that were matched in format and difficulty. Children between the ages of 5–12 years performed categorization tasks that targeted their ability to discriminate facial identity and facial expression. For both accuracy and reaction time, there was a main effect of age, and the improvement in performance on both tasks appeared steady from age five to twelve. There was no main effect of task and there was no interaction between age and task, indicating that both tasks showed approximately the same rate of improvement with age. These results indicate that these tasks are relatively well matched in terms of their sensitivity to developmental differences, suggesting that they may be useful for investigating individual differences in identity and expression processing (e.g. large dissociations in performance in individuals may indicate clinically significant impairments, such as prosopagnosia or autism). Importantly for our primary research question, these results appear to support the idea that face identity and expression processing share common mechanisms. However, they do not rule out the possibility that identity and expression processing are separable: it is still possible that two independent systems could develop at the same rate.

While an interaction between age and task would have provided compelling support for separable identity and expression processing systems, the lack of interaction in this study could be related to certain characteristics of our experimental design. First, given the finding that discrimination of face identity and memory for face identity develop at different rates [28], it is possible that tasks with a more demanding memory load could reveal different developmental trajectories for identity and expression processing. Our tasks are well-matched discrimination tasks, and the memory load is minimal: participants need only remember a single face, and the delay between the target and choice faces is brief (400 ms). Our study represents a first step towards directly comparing the development of identity and expression processing, but future experiments could manipulate memory load to further investigate the relative development of these two abilities. Second, these results could be a function of the age range tested. We tested pre-adolescent children 5-12-years-old, but others have found two separate stages in the development of facial expression recognition: one from 5–12 years, and another from 13 years through adulthood [47]. Testing children at intermediate ages (e.g. 9–16 years) with our matched tests may uncover differences in development that were not revealed here. Third, it is possible that rather than tapping identity and expression processing, our tasks in fact measure basic perceptual mechanisms that are not specific to either ability. We think this is unlikely since both tasks have a delay between the target face and the choice faces, preventing participants from engaging direct comparison or feature matching.

One related question about the present findings is whether the age-related improvements in identity and expression processing that we documented are face-specific (i.e. reflect the development of face processing mechanisms), or due to general cognitive development (i.e. reflect improved attention and other test taking skills). Unfortunately our study does not allow us to differentiate between the two alternatives.

This study does have important strengths: we tested a large number of children (over 100) from 5-12-years-of-age and our tasks are well matched in terms of format and difficulty, positioning them to reveal individual differences in identity and expression processing. This study represents a first step towards investigating the relative development of identity and expression processing, but these results do not preclude the possibility that the development of these abilities is indeed separable. More work needs to be done to address this issue. In the mean time, applying the present tasks to work with special populations (e.g. prosopagnosia, autism), may be useful for assessing whether, and at what developmental stage, one ability is disproportionately affected relative to the other in some individuals.

## Supporting information

S1 Fig Comparison of slopes for the two types of expression morph continuums across age. We compared the mean slope for Angry/Happy morphs to the mean slope for Disgusted/Surprised morphs using a 2-way ANOVA (Age x Expression type). We found a significant main effect of Age, F(1,28) = 90.4, *p*<0.001, and a significant main effect of Expression Type, F(1,28) = 16.5, *p*<0.001, but no Age x Expression interaction F(1,28) = 0.7, *p* = 0.40.
(TIF)

S2 Fig Individual accuracy data. Individual data representing accuracy for the Identity and Expression tasks, plotted by participant age.
(TIF)

S3 Fig Correlations: Accuracy. Correlation between accuracies on Identity task and Expression task plotted by participant age. Error bars represent 95% confidence intervals.
(TIF)

S4 Fig Correlations: Reaction time. Correlation between reactions times on Identity task and Expression task plotted by participant age. Error bars represent 95% confidence intervals.
(TIF)

S1 File Data. Raw data in.csv format.
(CSV)

S2 File Data description. Text file describing the data set.
(TXT)
