# Supplementary figures and images for: Concurrent development of facial identity and expression discrimination
Source: PLoS One. 2017 Jun 15;12(6):e0179458. doi: 10.1371/journal.pone.0179458 (PMC5472318; doi:10.1371/journal.pone.0179458)

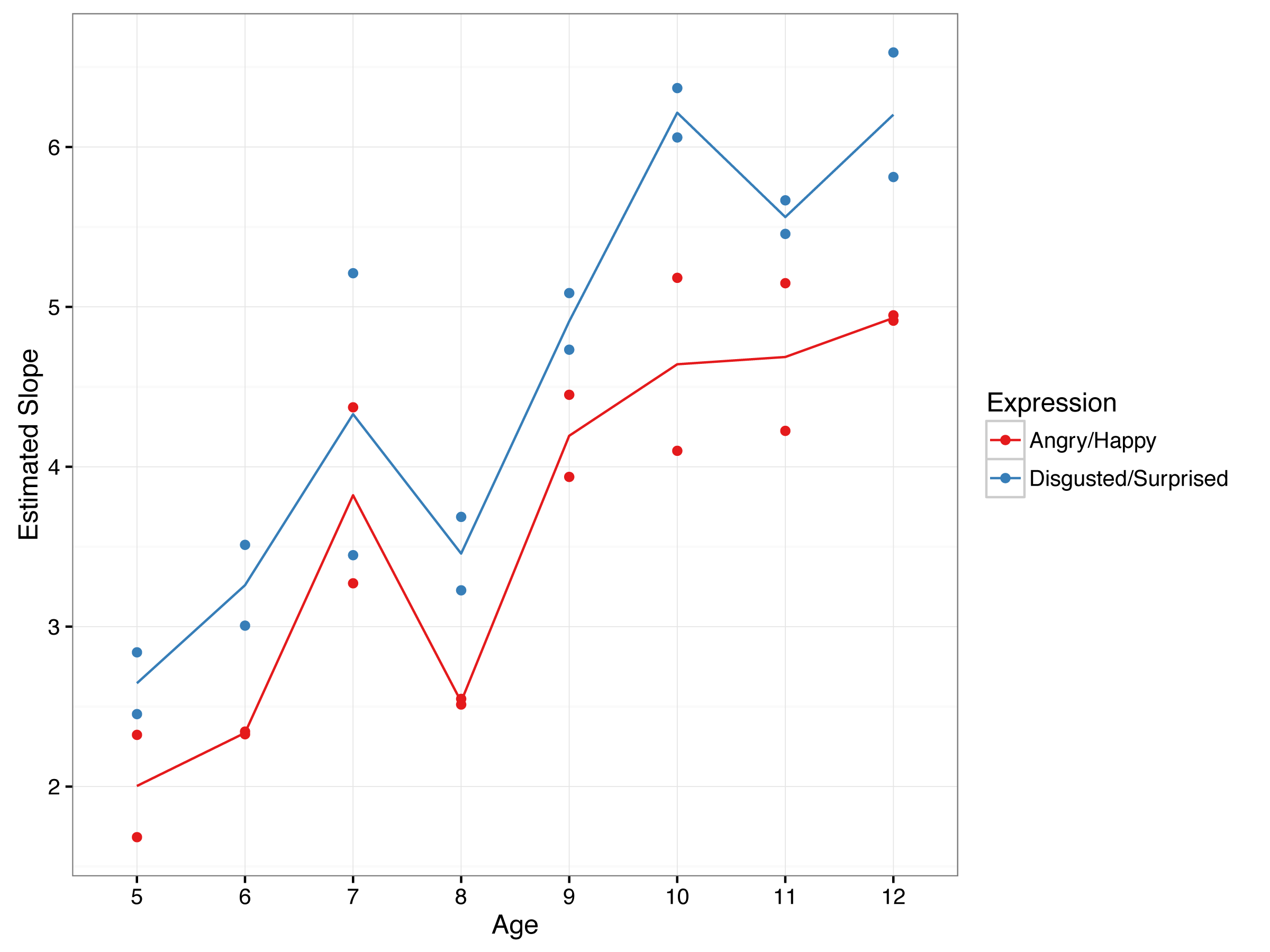

Supplement: S1 Fig — We compared the mean slope for Angry/Happy morphs to the mean slope for Disgusted/Surprised morphs using a 2-way ANOVA (Age x Expression type). We found a significant main effect of Age, F(1,28) = 90.4, p<0.001, and a significant main effect of Expression Type, F(1,28) = 16.5, p<0.001, but no Age x Expression interaction F(1,28) = 0.7, p = 0.40. (TIF) [file pone.0179458.s001.tif]

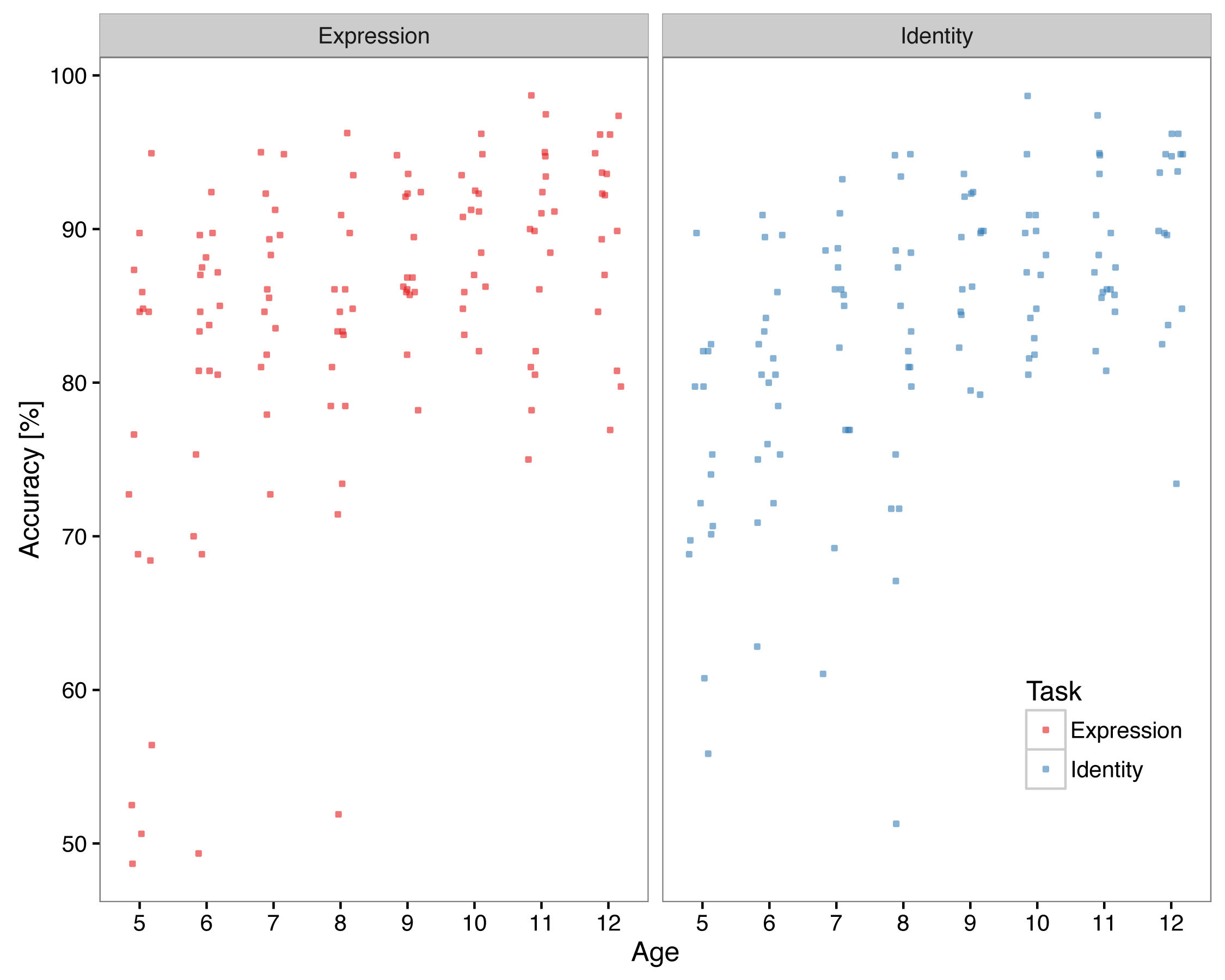

Supplement: S2 Fig — Individual data representing accuracy for the Identity and Expression tasks, plotted by participant age. (TIF) [file pone.0179458.s002.tif]

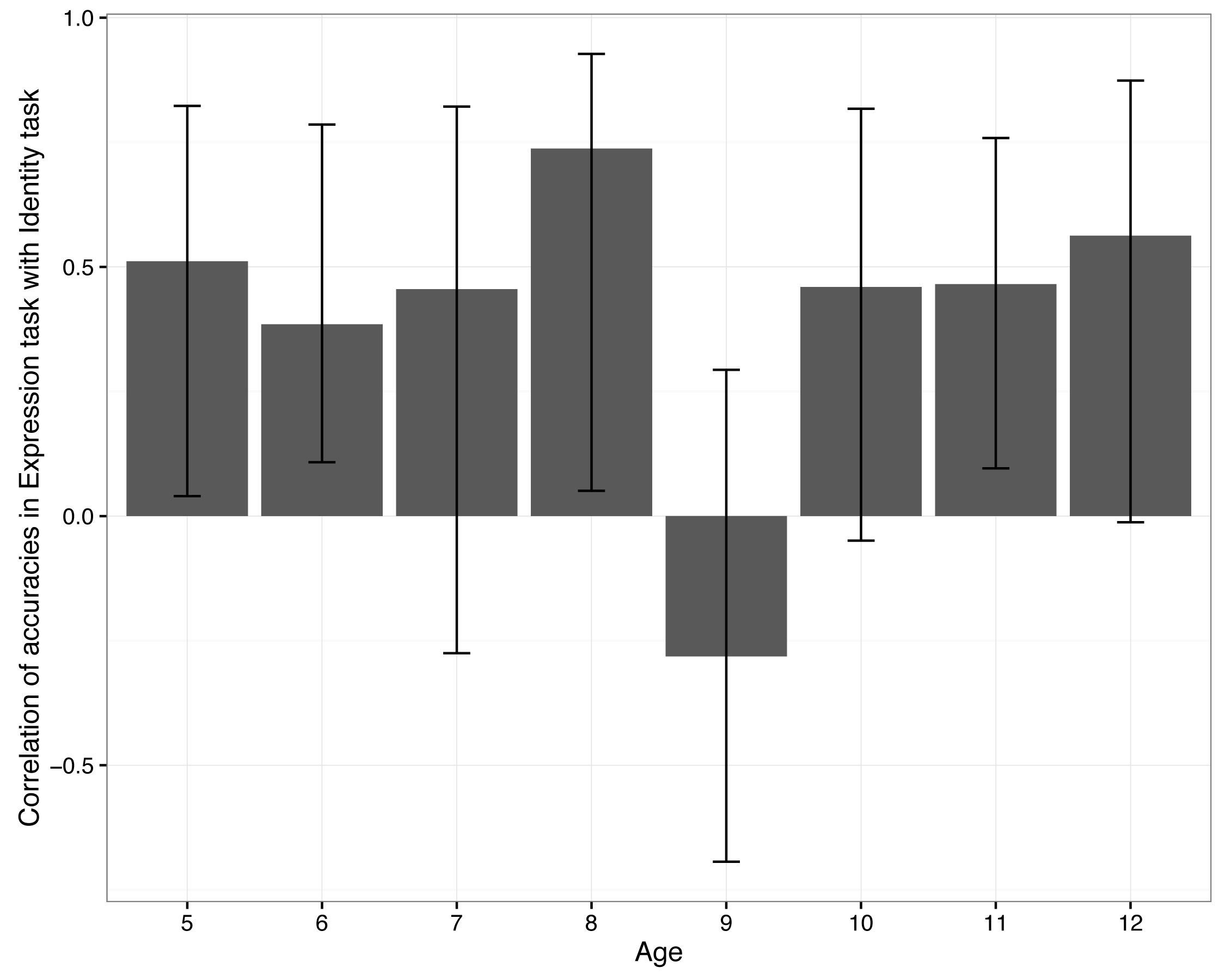

Supplement: S3 Fig — Correlation between accuracies on Identity task and Expression task plotted by participant age. Error bars represent 95% confidence intervals. (TIF) [file pone.0179458.s003.tif]

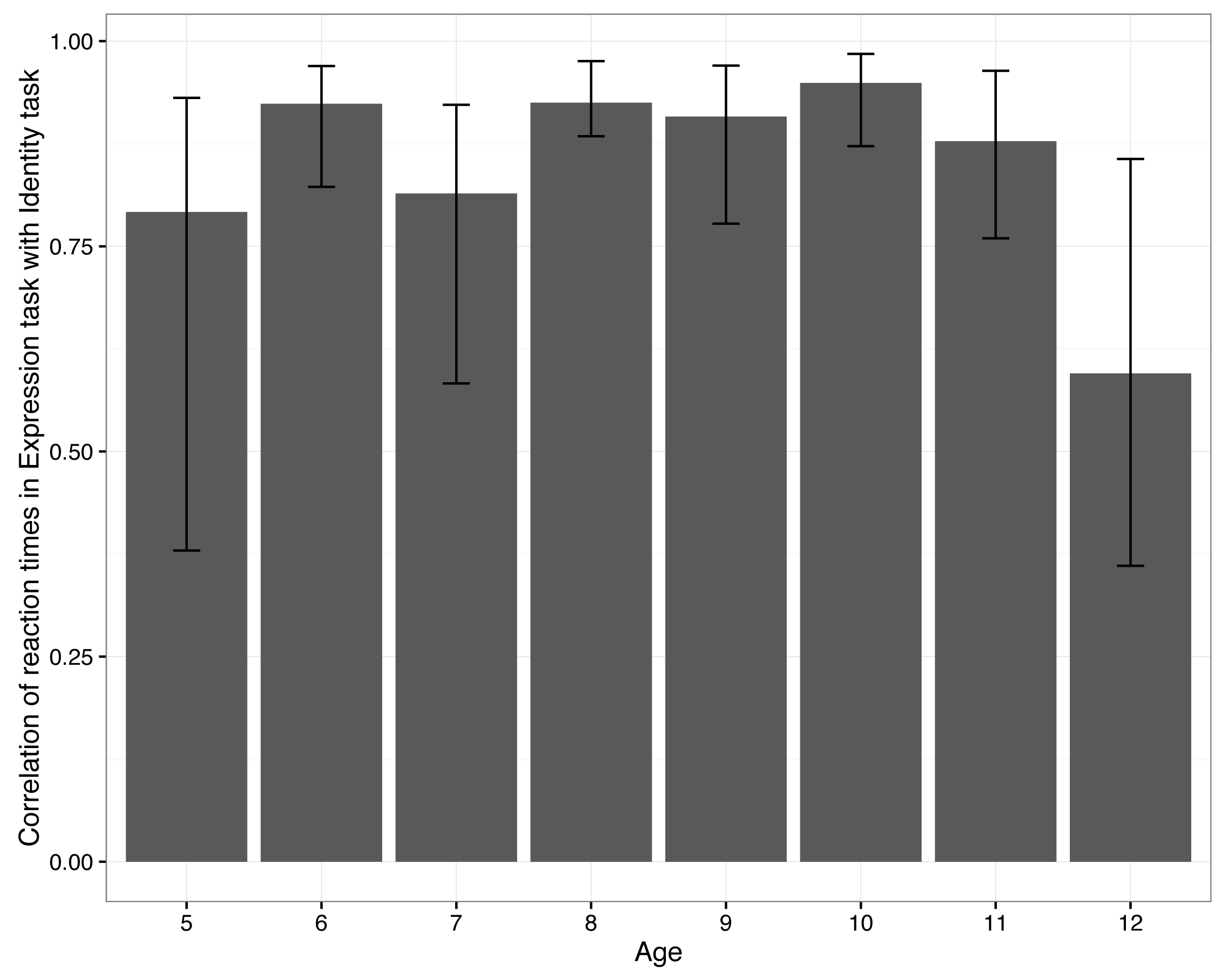

Supplement: S4 Fig — Correlation between reactions times on Identity task and Expression task plotted by participant age. Error bars represent 95% confidence intervals. (TIF) [file pone.0179458.s004.tif]
